# The Role of Non-Immune Cell-Derived Extracellular Vesicles in Allergy

**DOI:** 10.3389/fimmu.2021.702381

**Published:** 2021-08-19

**Authors:** Lilit Hovhannisyan, Ewa Czechowska, Danuta Gutowska-Owsiak

**Affiliations:** ^1^University of Gdansk, Intercollegiate Faculty of Biotechnology of University of Gdansk and Medical University of Gdansk, Gdansk, Poland; ^2^Department of in vitro Studies, Institute of Biotechnology and Molecular Medicine, Gdansk, Poland; ^3^Radcliffe Department of Medicine, University of Oxford, Oxford, United Kingdom

**Keywords:** extracellular vesicles, exosomes, cellular communication, immune responses, allergy, asthma, atopic dermatitis, allergic rhinitis

## Abstract

Extracellular vesicles (EVs), and especially exosomes, have been shown to mediate information exchange between distant cells; this process directly affects the biological characteristics and functionality of the recipient cell. As such, EVs significantly contribute to the shaping of immune responses in both physiology and disease states. While vesicles secreted by immune cells are often implicated in the allergic process, growing evidence indicates that EVs from non-immune cells, produced in the stroma or epithelia of the organs directly affected by inflammation may also play a significant role. In this review, we provide an overview of the mechanisms of allergy to which those EVs contribute, with a particular focus on small EVs (sEVs). Finally, we also give a clinical perspective regarding the utilization of the EV-mediated communication route for the benefit of allergic patients.

## Introduction

During evolution multicellular organisms developed diverse methods of communication including a direct cell-to-cell contact, which allows for receptor-ligand interactions as well as the release of active mediators providing intercellular information transfer between donor to recipient cells. These include both soluble molecules and extracellular vesicles (EVs) capable of travelling long distances within the body. EVs which comprise apoptotic bodies (AP; 100-5000 nm), ectosomes or shedding microvesicles (MV; 100–1000 nm), secreted mid-body remnants (sMB-Rs; 200-600 nm) and exosomes (50–150 nm) are a group of heterogeneous structures (1, 2) surrounded by a lipid bilayer. EVs are released from practically all cell types including epithelial cells, fibroblasts, mesenchymal cells, dendritic cells (DCs), B cells, T cells, mast cells and tumor cells, among others. The presence of EVs has also been shown in multiple body fluids, including saliva (3), plasma (4, 5), breast milk (6), urine (7), bronchoalveolar lavage (8, 9) and malignant effusions (10–12). The complete biological effects of EVs are not yet well understood, but it is known that MVs and exosomes can bind to cells through several mechanisms, including receptor-mediated endocytosis, direct fusion, phagocytosis, and caveolae- or clathrin-mediated endocytosis and transfer their content to the recipient cell (1, 13). It has also been shown that alveolar epithelial cells internalize MVs *via* fluid-phase endocytosis but not *via* the well-known receptor-mediated EV endocytosis (14); MV uptake has endocytic basis which is energy-consuming and requires cytoskeletal rearrangement (15); receptor-mediated MV uptake has also been reported (16). Because of their morphological characteristics, exosomes are considered the EVs most pronouncely involved in the information exchange process. The uptake results in functional effects in recipients; hence EVs contribute to the complexity of communication stream between distant cells. Besides the size and density, EV heterogeneity also derives from their diverse cargo, making it arduous for researchers to determine their exact functions (17).

Given their ability to regulate physiological and pathological processes (18, 19) there is growing interest focused on the potential of EVs to serve as novel targets for the development of therapeutic and diagnostic strategies. The role of different EV subtypes largely depends on the type and activation state of a cell producing them (20). Exosomes have been found useful in diagnostics as possible biomarkers, e.g. in oncology and nephropathies (21, 22) and as novel therapeutic approach for treating various diseases, including those with a clear immunological pathomechanism, e.g. atopic dermatitis, asthma, arthritis (23–25). In those, EVs produced by the immune cells are the main focus of the EV field. However, multiple kinds of non-immune cells, often overlooked, have been shown as efficient EV sources; these are often significant contributors to the ongoing immune response. This review, therefore, discusses the role of non-immune cell-derived EVs in immune processes in allergy in contrast to the immune cell-derived EVs.

## Extracellular Vesicles: Types and Biogenesis

**Figure 1:**
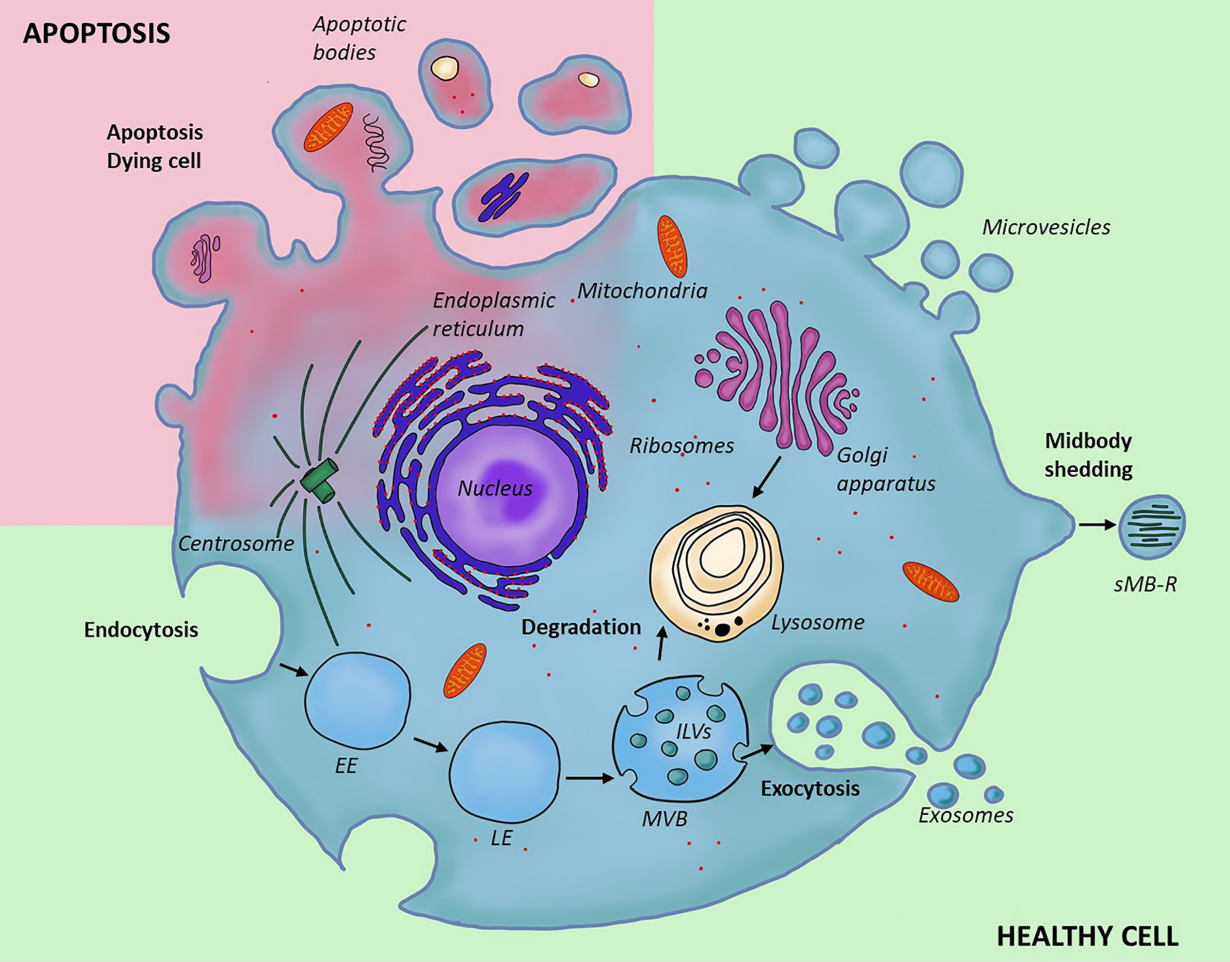
Different types and biogenesis of extracellular vesicles. Two types of EVs form through outward invagination of the plasma membrane; microvesicles and apoptotic bodies. The apoptotic bodies are larger and form in the context of programmed cell death; they enclose organelles removed from the cell during degradation, while microvesicles are produced by a healthy cell; their content is similar to that of the cytoplasm. Secreted midbody remnant is also secreted from the plasma membrane, but contain residual secreted midbody remnants are following cell division. In contrast to this, exosomes form through a distinct cellular pathway and within the endocytic system where inward budding of late endosome leads to the formation of a multivesicular body containing multiple intraluminal vesicles. The content of multivesicular bodies is either digested after fusion with lysosome (degradative pathway) or released into the extracellular space (secretory pathway). EE, early endosome; LE, late endosome; MVB, multivesicular body; ILVs, intraluminal vesicles; sMB-R, secreted midbody remnant.

EVs are most frequently categorized based on their biogenesis, and sub-grouped into three major types: exosomes, **microvesicles and apoptotic bodies** (**Figure 1**). Recently, a novel type of EVs, namely **secreted midbody remnants (sMB-Rs)** have also been described, along with yet another type of secreted nanoparticles, i.e. “exomeres”. While the former appear to be membranous structures and are likely true vesicles, a debate on the latter is ongoing (due to the lack of consensus we did not include exomers in **Figure 1** and **Table 1**). The differences in the origin are directly reflected in the variations in the size, morphology, cargo and surface content of those EV populations (**Table 1**); however, they all likely play a role in cell-to-cell communication, transferring a variety of biological molecules, i.e. proteins, lipids, nucleic acids and small molecular mediators (18, 19, 75–80) to the recipient cell.

**Table 1:** Common markers and cargo found in EVs.

EV type	Markers	EV Cargo
**APs** (100-5000 nm)	Phosphatidylserine (26) TSP (27) C3b (28) Calreticulin (29)	DNA (30, 31) RNA (32) Peptides (31) Phospholipids (31) Annexin V (31) Lipids (33)
**MVs** (100-1000 nm)	Actinin-4 (34) Integrins (35) Selectins (36) Flotillin-2 (37) CD40 ligand (36) Metalloproteinase (38) ARF6 (39) VCAMP3 (40) KIF23 (41)	DNA (42) RNA (32, 43) Poteins (44) Receptors (45–48) Lipids (49)
**sMB-Rs** (200-600 nm)	KIF23 (2, 50, 51) Prominin-1 (52)	Proteins (2) Centraspindlin (2)
**Exosomes** (50-150 nm)	CD81 (53) CD82 (53) CD9 (54) CD63 (55, 56) Alix (54, 57) TSG101 (57) Flotillin-1 (58, 59) Syntenin (34) Hsp70 (60) CD24 (61)	Receptors (62, 63) Cytoplasmic proteins (64, 65) Tetraspanins (66) DNA (67) RNA (68, 69) Lipids (70) MHC complex (71, 72) Integrins (73) Cytoskeletal components (74)

**Exosomes** compose a population of small EVs (50-150 nm) (1). Due to their size and composition mainly consisting of lipids, these vesicles can squeeze between cells without damage and enter the circulation; this facilitates transfer of their cargo between cells at the longest distances (55, 81, 82). The exosomal wall composition reflects the biogenesis of those vesicles which have unique endocytic origin. Specifically, exosomes form at the level of late endosomes (LEs) which later progress into multivesicular bodies (MVBs) by accumulation of intraluminal vesicles (ILVs) generated through inward budding of the LE membrane (83). The formation of MVBs is mediated by two separate pathways; one involving a multimolecular machinery called *endosomal sorting complex required for transport* (ESCRT) and the other, dependent on a specific lipid composition of the endosomal membrane (84). ESCRT is a protein cascade consisting of approximately 30 proteins which are integrated into four subunits, namely ESCRT-0, ESCRT-I, ESCRT-II and ESCRT-III (83, 85, 86). The role of ESCRT-0 is to recognize and sequester ubiquitinated transmembrane proteins in the endosomal membrane which allows the ESCRT-I to bind to these ubiquitinated proteins and activate ESCRT-II to start oligomerization and generation of ESCRT-III. ESCRT-I and ESCRT-II complexes are implicated in the process of membrane deformation which leads to the membrane budding, and ESCRT-III components accomplish vesicle scission (1, 44, 87–89); the ESCRT pathway is ATP-dependent. To disassemble ESCRT subcomplexes from the endosomal membrane, the AAA (ATPases Associated with diverse cellular Activities); ATPase VPS4 (Vacuolar Protein Sorting 4), is required, which enzymatically accomplishes the membrane abscission (90–92). During MVB sorting an accessory factor, ALIX, is required for exosome secretion at the endosome to help sort membrane proteins into vesicles which later bud into MVBs (93, 94). Larios et al. have shown that ALIX- and ESCRT-III–dependent pathway promotes sorting and delivery of exosomal proteins (95). In contrast, the ESCRT-independent pathway relies on the process of converting membrane sphingolipids to ceramides by sphingomyelinase which is necessary for the inward budding and formation of ILVs (57, 96–98). Following the budding, MVBs which accumulate ILVs either fuse with the plasma membrane to release exosomes into the extracellular space *via* exocytosis (secretory pathway) or fuse with lysosomes and their content is digested by the lysosomal enzymes (degradative pathway) (99–101). The ESCRT-independent formation of ILVs in MVBs has been shown to be regulated by CD63 tetraspanin, which is particularly enriched intracellularly and is mostly localized in the endosomes and lysosomes, although in specialized cells it is also associated with lysosome-related organelles and their endosomal precursors (102, 103). Edgar et al. have shown that the formation of small ILVs requires CD63 (104).

**Microvesicles** (MVs) are vesicles generally larger than exosomes, with sizes in the 100-1000 nm range but some smaller MVs may be difficult to distinguish from exosomes purely based on the size. However, their biogenesis is completely unrelated; they originate through the processes of direct outward budding and fission of the plasma membrane into the extracellular space (105, 106); this explains why the MV surface markers largely depend on the composition of the plasma membrane (107). Based on the way of how the plasma membrane has emerged during the MV formation, MVs may contain various cell surface proteins, such as ARRDC1 (arrestin domain-containing protein 1) (108, 109), Bin-1 (ampiphysin) (110), EGFR (epidermal growth factor receptor), etc. (111). Released MVs may be taken up *via* receptor-mediated uptake (16, 112, 113) to transfer their cargo (surface receptors, lipids, proteins, mRNA, miRNA, infectious particles e.g. prions) to the target cells.

**Apoptotic bodies** (APs) are the largest subfraction of extracellular vesicles (100-5000 nm), formed and released when the cell undergoes programmed cell death, i.e. apoptosis (114, 115). Many changes occur to the cell during this process, including pronounced changes to the plasma membrane. Specifically, the blebbing generates various types of protrusions and APs form and may be released from those (116, 117). APs carry antigens and a variety of biomolecules, intracellular fragments, disrupted and degraded cellular organelles, membranes, released nucleic acids and cytosolic contents (75). APs have been shown to transfer their cargo and content between various cells (30, 118). Interestingly, the communication with the immune cells specifically is commonly mediated by vesicle-associated cytokines or damage-associated molecular patterns (DAMPs) (119). This includes mitochondria-derived N-formylated peptides (120), the nuclear protein High Mobility Group Box 1 (HMGB1) (121), histones (122), calcium-binding S100 proteins (123), heat shock proteins (HSPs) (124), ATP (125), uric acid (126), DNA/RNA and actin among many others (127). This richness of the cargo is perhaps not surprising, given the context of the cell death resulting in AP formation. Immune cells recognize these molecules *via* pathogen recognition receptors (PRRs) and drive inflammatory responses (117, 128, 129). APs act locally and are removed from the extracellular environment during phagocytosis by macrophages (117, 130, 131).

Very recent developments in the EV field brought identification of two new types of nanoparticles, i.e. exomeres and secreted midbody remnants. Given their novel nature, these are not yet well described, both in relation to their structure and function, however certain aspects are already known which positions these nanoparticles in the interest of the EV field.

**Secreted midbody remnants (sMB-Rs),** with sizes in the 200-600 nm range have been described as particles generated during the cell division. Specifically, these are generated at the time when daughter cells are still connected with intercellular cytoplasmic bridge; this bridge is cut during the cytokinesis by a transient organelle called *midbody* which anchors SNARE and exocyst complexes (50, 132). As a consequence, one of the nascent cells retains these midbody remnants and discards them either by autophagy (133) or releases them in the form of secreted vesicles; sMB-Rs. It has been documented that these nanoparticles are distinct from exosomes and shed microvesicles (51, 52). While generated as a byproduct during the cell division, sMB-Rs may also convey messages when internalized, as shown for fibroblasts, in which sMB-Rs promote cellular transformation into an invasive phenotype (2).

**Exomeres,** with their size at the ≤50 nm mark are the smallest secreted nanoparticles described so far. They also have very distinct characteristics; of all, the lack of a limiting membrane is the most evident differential feature. Exomeres seem to be involved in cargo transport and have been shown to contain proteins, lipids and nucleic acids, which provide functional outcome by receiving cells. Currently, however there is a debate whether exomers should be classified as “vesicles” and the EV field is awaiting specific recommendations in this regard (134–136).

It should be noted that while distinct types of EVs can be described by their origin pathway, as well as a set of specific characteristics including the size, marker profile and cargo content, the technical caveats and lack of very unique markers available to unambiguously define every EV population, it is virtually impossible to specify the origin of the EVs, unless these are imaged during secretion. Therefore, following the recommendation of the International Society for Extracellular Vesicles (ISEV) for the purpose of this review we have used the terms “small” and medium/large EVs” (sEVs and m/lEVs) throughout instead of the original description published in the referenced papers unless the populations are very well defined according to the ISEV guidelines (137).

## Immune Cell-Derived EVs in Immunity

Recent progress in the EV field determined that thorough understanding of the EV biology and function is pivotal for our comprehension of immune-driven diseases, including the pathogenesis of allergy. Here, immune cell-derived EVs emerge as important contributors to immune responses, in both the innate and adaptive immunity arms and it may be useful to explore their potential as diagnostic and therapeutic tools. In the innate immunity pathways EVs provided by NK cells, macrophages and neutrophils mediate early host recognition and elimination of invading pathogens. In the adaptive arm, EVs are capable of activating B cells for antibody responses as well as providing both the direct and indirect antigen-specific stimulation to T cells. For the former, class I and class II MHC molecule-enriched EVs from antigen-pulsed DC are able to act as a display system for antigen presentation to cytotoxic and helper T cells (138). Moreover, it has been shown recently that the responses induced by exosomes (defined as tetraspanin and syntenin-positive sEVs) are by far superior in comparison to those obtained from MVs (distinguished as actinin-1-positive, syntenin-negative) (34), further highlighting the distinctive features resulting from the unique exosomal biogenesis pathway, encompassing the MHC-reach cellular compartments. As far as the indirect presentation is concerned, antigen or antigen/MHC complex transfer is also engaged, as well as cross-priming and cross-dressing presentation pathways. These topics have been extensively covered already in excellent publications (138–144). Hence, since the focus of this review is the EVs secreted by non-immune cells which are often overlooked but also extensively participate in immune responses, their contribution will be presented next.

## Non-Immune Cell-Derived EVs in Immunity

**Figure 2:**
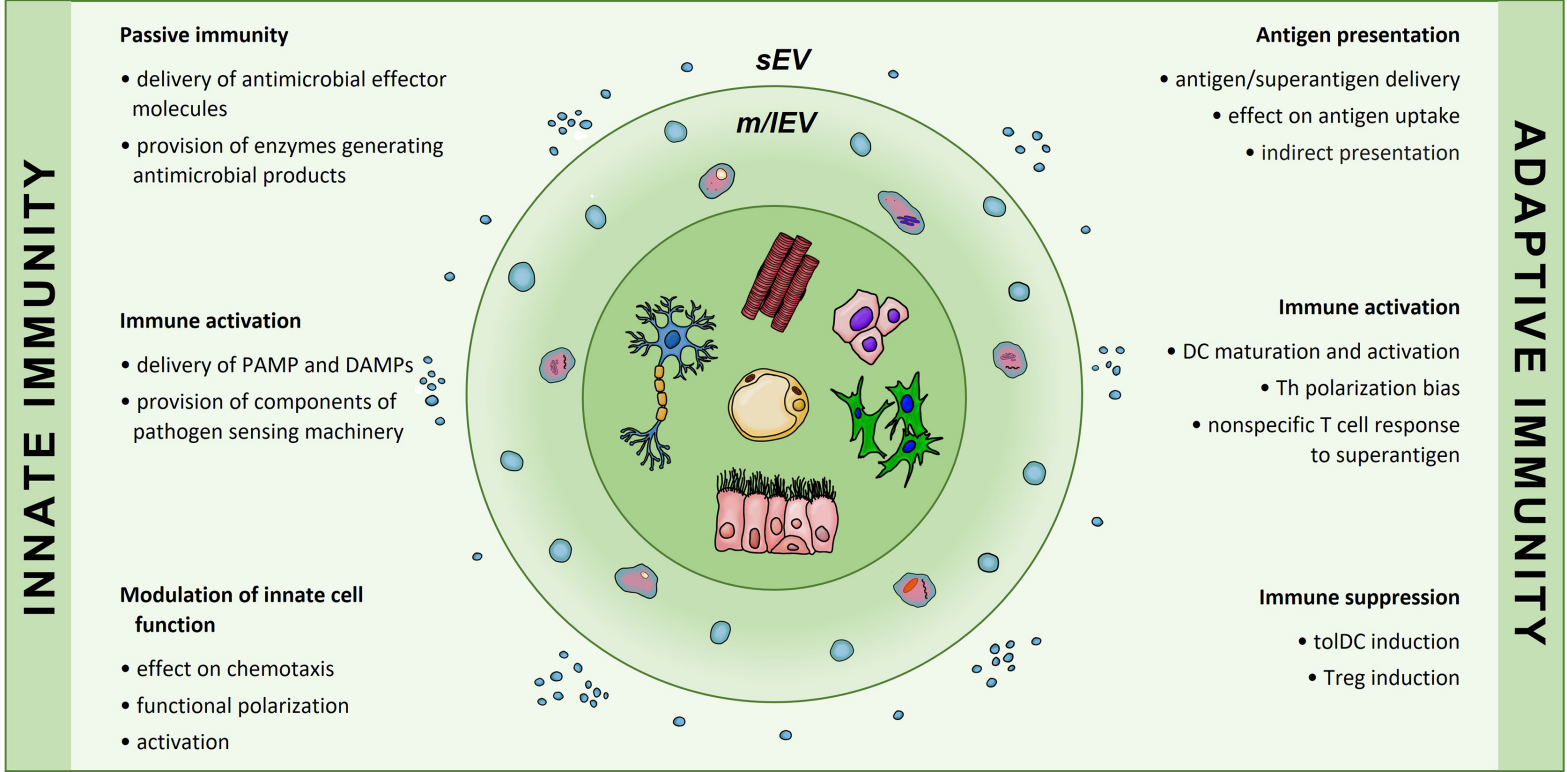
Involvement of non-immune cell-secreted extracellular vesicles in immunological processes of innate and adaptive immunity. Extracellular vesicles produced by cells of non-immune origin participate in exchange of information that contributes to immune responses. In the innate arm EVs enable passive immunity and may both induce activation and modulate innate cell function. In the adaptive arm EVs may influence antigen presentation, affect dendritic cell differentiation and phenotype; they have also been implicated in T cell polarization into Th or Treg subsets. sEVs, small EVs; m/lEVs, medium/large EVs.

While not as potent, in some respects, as the EVs secreted from the immune cells, the EVs that are produced by the non-immune cell types have also been shown to exert many distinct roles in the immune system. These non-immune EV-mediated pathways include contribution to both innate and adaptive immunity, ranging from the activating to inhibitory roles (**Figure 2**). As with any cells, the relative impact depends on the type and the activation state of the donor cell, in parallel to the functionality observed at the cellular level. Next section will discuss the ways in which those non-immune cell-derived EVs participate in the mechanisms of the innate and adaptive immunity.

### Non-Immune Cell-Derived EVs in Innate Immunity

EVs secreted by non-immune cells provide mechanisms of innate control and consist a link between innate immunity and allergic diseases (145). For example, Hu et al. have shown that the activation of the TLR4 signaling results in enhanced luminal sEV production and shuttling of the epithelial antimicrobial peptides (cathelicidin-37 and β-defensin 2) from the gastrointestinal epithelium (146). Nasal mucosa-derived sEVs were also shown to carry proteins involved in the innate immune responses, including inducible nitric oxide synthase (NOS2) which exerts antimicrobial function (147). Similarly to this, Nocera et al. have shown that the secretion of basal nasal mucosa-derived sEVs and the expression of exosomal NO is increased after TLR4-stimulation by lipopolysaccharide. Interestingly, mucosa-derived sEVs had microbiocidal properties and were capable of transferring their immunoprotective cargo to naive epithelial cells to confer passive immunity to recipient cells in the setting of chronic rhinosinusitis (148).

Non-immune cell-derived sEVs can also interfere with the NOD-dependent signaling. Specifically, Vaccari et al. have shown that the expression of the components of the nucleotide-binding-and-oligomerization domain (NOD)-like receptor protein-1 (NLRP-1) inflammasome are increased in the spinal cord motor neurons and cortical neurons after trauma. Interestingly, NLPR-1 inflammasome proteins were found in cerebrospinal fluid-derived sEVs after spinal cord injury and traumatic brain-injured patients. The authors have shown that sEVs derived from neurons loaded with short-interfering RNA against caspase recruitment domain (CARD) can deliver their cargo and reduce inflammasome activation following spinal cord injury in rodents (149). Following this, Li et al. have demonstrated the ability of hepatocyte-derived sEVs (expressing exosome-associated tetraspanins) to induce acute liver injury in severe heat stress by activating the NOD-like receptor signaling pathway in hepatocytes (150). This pathway seems to provide a link between visceral organs and the central nervous system (CNS) as shown in a hepatic ischemia-reperfusion injury model. Liver transplantation may result in neuronal injury and cognitive dysfunction (151); Zhang et al. have demonstrated that circulating sEVs play critical role in hippocampal and cortical injury through regulating neuronal pyroptosis in rats. The authors have shown that neuronal pyroptotic cell death may be caused by sEVs through TLR4 activation of NLRP3 inflammasome (152).

Exosomal transfer of pathogen recognition pathway components may convey the message to the immune cells, such as monocytes and macrophages. Specifically, Mills et al. have shown that poly(I:C) stimulation induces the release of tenascin C-rich sEV from airway epithelial cells; these may potentiate airway inflammation by promoting cytokine production in macrophages (153). Furthermore, airway epithelial cell-derived sEVs have been shown to induce proliferation and infiltration of undifferentiated macrophages into the lungs under the influence of IL-13 in a murine model (20). In contrast, mesenchymal stem cells (MSC)-derived sEVs are capable of inhibiting macrophage chemotaxis (154), altering the M1/M2 balance (155), inhibiting M1 *via* miR-147 (156) and stimulating the M2 polarization in monocytes (157).

### Non-Immune Cell Derived-EVs in Adaptive Immunity

Multiple studies indicate that non-immune cells, in the steady state, secrete EVs that execute immunoregulatory roles in adaptive immunity. For example, bone marrow MSC-derived EVs have been shown to suppress the Th2/Th17-mediated airway hyperresponsiveness and lung inflammation in a model of *Aspergillus hyphal* extract-induced allergic airway inflammation (158). Indeed, MSC-derived sEVs have shown immunosuppressive effects on several types of immune cells (159); including inhibition of B cell and DC proliferation (25), B cell maturation (160), and induction of T regulatory cells (Treg) (161–163). More specifically, Gomzikova et al. have demonstrated that MSC-derived EVs alter DC maturation and functional state (164); the antigen uptake by immature DCs was attenuated and the stimulation rendered DCs with a semi-mature phenotype after LPS exposure (165). These phenotypic changes were accompanied by a functional shift in the cytokine production profile from inflammatory to immunoregulatory (164), suggesting that those sEVs could promote tolerogenic DC (tolDC) induction. MSC-derived EVs have been also shown to reduce inflammatory cytokine (IL-23 and IL-22) production (166), enhancing the anti-inflammatory phenotype and regulatory lymphocyte proliferation, and the ability to produce IL-10 and TGF-β (167). Proliferation of T cells has also been shown to decrease after MSC-derived EV treatment *in vitro*, accompanied by a downregulation in IFN-γ and TNF-α (168). The study by Shigemoto-Kuroda et al. also confirmed that MSC-derived EVs have the ability to suppress Th1 and Th17 development, inhibit antigen presenting cell activation and increase expression of the immunosuppressive cytokine IL-10 (169). In a limited model, murine epidermal keratinocyte-derived sEVs (flotillin and Alix-positive) failed to induce T cell immune response despite some phenotypic effects on DC (170). However, when the donor cells are subjected to IFN-γ activation, keratinocyte-derived sEVs (of exosomal marker characteristics) may act as a transfer vehicle for T cell stimulation by *Staphylococcal aureus* enterotoxin B. Specifically, in this context HaCaT keratinocytes were shown to produce sEVs that contain MHC class I and class II and were able to drive nonspecific proliferation of CD4^+^ and CD8^+^ T cells *in vitro* (171). This suggests that the relative contribution of non-immune cell-derived sEVs (and potentially other EVs) to the adaptive immunity and T cell reactivity may change depending on the stimulation received by the donor cell; further evidence supports this (172).

Interesting are the results by Admyre et al. who have demonstrated that human breast milk contains sEVs which reveal immunomodulatory features inhibiting T cell cytokine production from PBMC and increasing the number of Foxp3^+^CD4^+^CD25^+^ Tregs in this semi-allogenic system (173, 174). Based on the content of surface molecules, in comparison to the DC-derived sEVs, these sEVs originate from either macrophages and lymphocytes in the breast milk or rather breast epithelial cells (173, 175). To support this, Herwijnen et al. have also shown that human milk-derived EVs contain novel EV-associated bioactive proteins that have distinct functions from other milk proteins; this suggests a novel mechanism of cellular communication between the mother and newborn (176).

## The Role of Non-immune Cell-Derived EVs in Allergic Conditions

**Figure 3:**
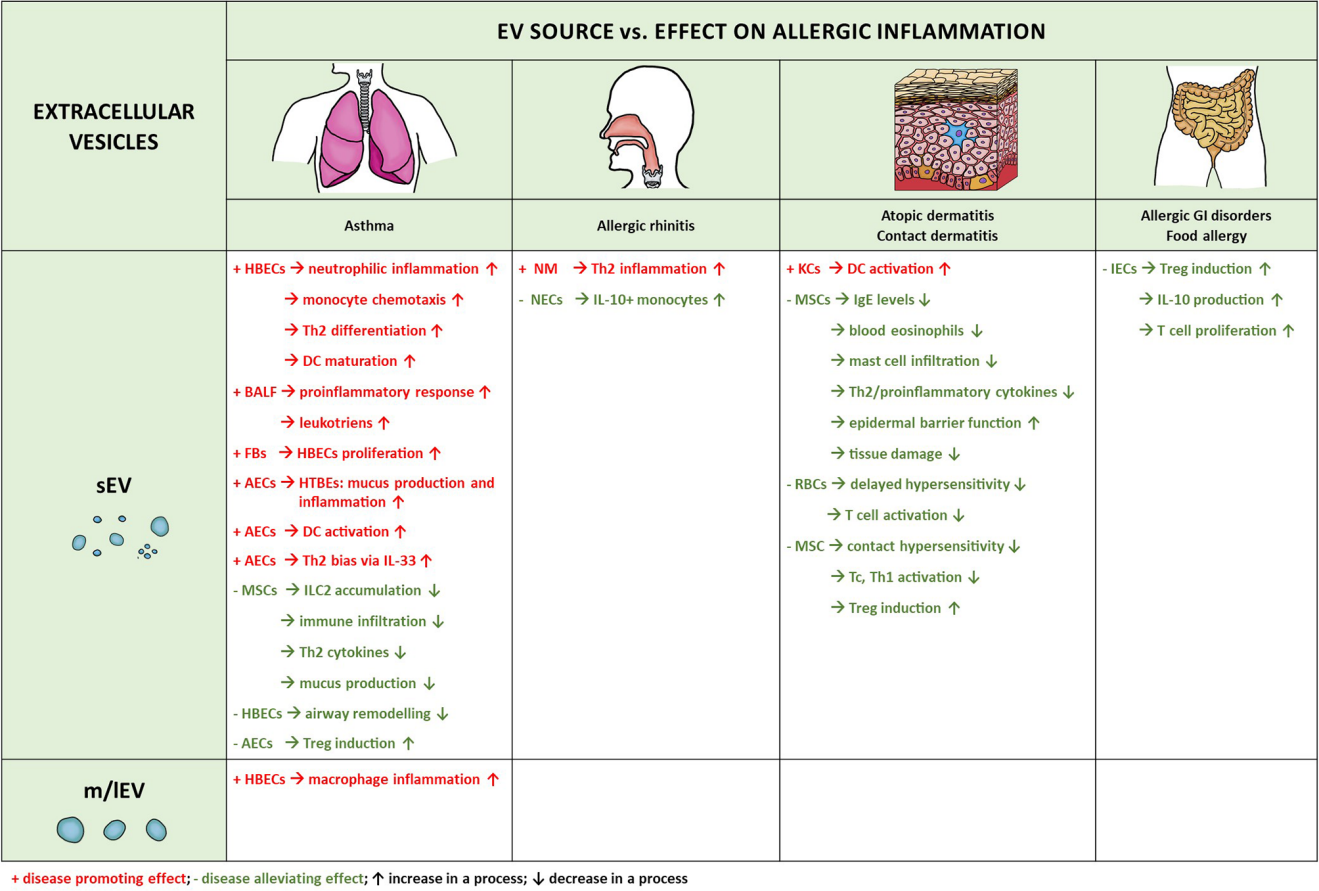
Extracellular vesicles produced by non-immune cells and their involvement in allergic diseases. Microvesicles and exosomes are the two types of extracellular vesicles which have been implicated in the pathogenesis of allergic inflammation. There is significant predominance of the exosomal involvement, likely due to the phenotypic characteristics and physical properties of these vesicles, enabling more without damage and entering the circulation for long-distance delivery. HBECs, human bronchial epithelial cells; BALF, bronchoalveolar lavage fluid; NM, nasal mucus; NECs, nasal epithelial cells; AECs, airway epithelial cells; HTBEs, human tracheobronchial cells; RBCs, red blood cells; IECs, intestinal epithelial cells; KCs, keratinocytes; FBs, fibroblasts; MSCs, mesenchymal stem cells. ↑ increase in a process; ↓ decrease in a process; + disease promoting effect; - disease alleviating effect.

Many studies have been performed to investigate the involvement of non-immune cell-derived EVs content/cargo with different clinical manifestations of allergy; in this section current research regarding the role and function of non-immune cell-derived EVs in allergic conditions is reviewed. Certainly, for the outcome in allergic inflammation much depends on the source of EVs as summarized in **Figure 3**.

### Asthma

EVs contribute to the asthma pathogenesis *via* various mechanisms, related to both inflammation and pathological remodeling (177) and there are interesting interdependencies that can be observed. Specifically, it has been shown that fibroblasts-derived EVs secreted by cells obtained from severe asthmatics increase proliferation of bronchial epithelial cells (HBECs) in comparison to those in healthy individuals, due to a decrease in the TGF-β2 content (178). Vice versa, vesicular transfer between epithelial cells and fibroblasts which includes inositol polyphosphate 4-phosphatase type I A (INPP4A) cargo, may regulate inflammation and airway remodeling (179). Further to that, Gupta et al. have shown that sEV transfer between airway epithelial cells (AECs) and human tracheobronchial cells (HTBEs) promotes expression several proteins which may contribute to allergic inflammation and exacerbation of asthma symptoms, i.e. gel-forming mucins (180), complement component C3, SERPIN3. The addition of an allergen source (house dust mite; HDM) to the AEC culture resulted in DC activation by secreted sEVs *in vitro* and increased airway inflammation in a murine model (181); the role for contactin-1 has been demonstrated. Furthermore, it has been also demonstrated that sEVs may be a vehicle of secretion for an important Th2-promoting cytokine, interleukin 33 (IL-33); the cytokine seams to decorate the EV surface rather than be included within the intraluminal cargo (182). At the same time, however, it has been also shown that CD83/OVA-carrying sEVs derived from those cells may promote Treg differentiation (183). Ax et al. have documented that HBECs increase the number of EVs released upon treatment mimicking asthma *milieu* which may contribute to establishing of the neutrophilic airway inflammation associated with Th17-driven asthma (184). Kulshreshtha et al. have shown that IL-13-treated epithelial cells secrete sEVs which stimulate proliferation and chemotaxis of monocytes; suppressing secretion of those sEVs in the lungs alleviates asthmatic inflammation in a murine model of bronchial asthma (20). Similarly, Lee et al. have shown that HBEC-derived m/lEVs may promote macrophage-mediated inflammation upon hyperoxia-mediated lung injury *via* miR-221 and/or miR-320a (185). Moreover, three additional miRNAs, i.e. miR-92b, miR-34a and miR-210 found in the sEVs secreted by HBECs have been suggested to have possible roles in regulating Th2 differentiation and DCs maturation in asthma, indicating that airway epithelial miRNA secretion *via* sEVs might be even more implicated in the development of the disease (186). In agreement with this, bronchoalveolar lavage fluid (BALF)-derived EVs isolated from LPS-treated mice drive a mixed Th1/Th17 cell response and enhance production of the Th1/Th17-polarizing cytokines (IL-12p70 and IL-6) by lung DCs in an asthmatic mouse model but are more tolerogenic if the animals are devoid of the LPS stimulation (70). Specifically, Paredes et al. have shown that asthmatic BALF-derived sEVs which carry tetraspanins and MHC class II molecules might reflect increased levels of antigen-presenting capacity and suggest that these sEVs might contribute to the inflammation by increasing cytokine and leukotriene production in AECs (187). Asthmatic patients also have altered sEV proteomic characteristics and eicosanoid profile which is shown to exert pro-inflammatory functions *in vitro*. Specifically, Hough et al. have shown that BALF-derived EVs contain lipids, such as ceramides, sphingosines, prostaglandins and leukotrienes which have been previously identified to drive inflammation in asthma (188). In asthmatic conditions, BALF-derived EVs also exhibit particular miRNA profiles (189) and carry the biosynthetic machinery for the leukotriene biosynthesis pathway (187, 188). In agreement with this, in a human study, EVs isolated from the nasal secretions of children with asthma and chronic rhinitis promoted trafficking of primary monocytes, NK cells and neutrophils thanks to the changes in the exosomal proteome contributing to the alterations in the immune-related functions (147). These effects can be contrasted with a healthy lungs, as demonstrated in an animal model by Wan et al., who have shown that EVs isolated from the lungs of healthy mice contain immunosuppressive cytokines TGF-β1 and IL-10 which inhibit T helper cell proliferation and relieve asthmatic symptoms in mice (190). The immunomodulatory effect of EVs was also demonstrated by Prado et al. who have shown that intranasal administration of sEVs isolated from BALF of mice tolerized against major pollen allergen in the murine airway inflammation model (Ole e1) induces tolerance and protects naïve mice against allergic sensitization (191).

Finally, innate lymphoid cells type 2 (ILC2s) which are large contributors to the Th2-dominated allergic inflammation in the airways (192) can also be targeted by EV-mediated suppression. Specifically, systemic administration of MSC-derived sEVs resulted in the reduction in the ILC2 accumulation, inflammatory cell infiltration and mucus production in the lung, a reduction in the levels of Th2 cytokines, and alleviation of airway hyperresponsiveness in a mouse model of asthma. It seems that this sEV-mediated preventive effect was conveyed by the transfer of miR-146a-5p (193).

### Allergic Rhinitis

Allergic rhinitis (AR) is a disease manifesting as type I allergic hypersensitivity within the nasal mucosa (194), and is characterized by chronic inflammation (195). The imbalance between the Th1 and Th2 differentiation is involved in the development of AR which is suggested to be partly regulated by sEVs. Zhu et al. have reported expression of a long-noncoding RNA (Lnc) GAS5 in the nasal mucus-derived sEVs in AR and in the ovalbumin-stimulated nasal epithelial cell (NEC)-derived sEVs. Here, this Lnc RNA promoted suppression of Th1 cell differentiation and induced Th2 differentiation upon treatment with nasal mucus (NM)-derived sEVs. A potential mechanism seems to involve the regulation of Enhancer of Zeste Homolog 2 (regulating proliferation and differentiation processes, including mediating proliferation and apoptosis of allogeneic T cells), and inhibition of T-bet expression by long-noncoding RNA GAS5 (196).

NEC-derived exosomal miR-146a induces the expression of IL-10 in monocytes in the murine model which seems to suppress allergic reactions downstream. Specifically, IL-10^+^ monocytes have an immune suppressor effect on the CD4^+^ effector T cells and the Th2 polarization in this model of AR (197). Interestingly, the alterations in the miRNA profile obtained from NM-derived EVs of AR patients showed intrinsic dysregulation of EV miRNA content in the disease. Wu et al. have demonstrated significant enrichment of certain biological and cellular processes within these differentially expressed miRNA signatures, namely B-cell receptor signaling pathway, natural killer cell-mediated cytotoxicity and T-cell receptor signaling, among others, implying that vesicular miRNAs exert regulatory function in AR. When investigated in more detail, B cell receptor signaling pathway-related miR-30-5p and miR-199b-3p were significantly increased, also miR-874 and miR-28-3p were significantly down-regulated in EVs from nasal mucus in AR (198).

### Atopic Dermatitis and Contact Allergy

Atopic dermatitis (AD) is a chronic inflammatory skin disorder associated with the epidermal barrier disruption, eczematous cutaneous lesions and severe pruritus. AD pathogenesis is complex and characterized by cytokine production predominantly mediated by Th2 cells and ILC2 (199), but also involving innate and Th17 and Th22 components (200).

The importance of keratinocytes of the skin in the disease pathogenesis has been highlighted by the findings demonstrating that insufficiency in the epidermal barrier is key component (201). However, only one study so far has investigated the impact of EVs secreted by keratinocytes in the context of allergic inflammation (170). Here, using a murine allergy model, the authors noticed some signs of DC activation upon exposure to an antigen (OVA peptide) transferred by sEV from secreting keratinocytes. At the same time, however, they failed to detect any changes in the T cell reactivity to this peptide antigen.

Besides that, little is known about EV secretion from other cells in the skin with relation to AD, with more focus directed towards potential new therapies. In this regard, it has been reported that intravenous/subcutaneous administration of human adipose tissue-derived MSC-derived sEVs (showing exosomal characteristics) ameliorate AD symptoms *in vivo* (in a mouse model); the levels of serum IgE, the number of eosinophils in the blood, and the infiltration of mast cells were also shown to be reduced after the treatment. Such sEVs also reduced mRNA levels of IL-4, IL-31, IL-23, and TNF-α in the skin lesions demonstrating that their systemic administration may ameliorate AD-like symptoms through the regulation of inflammatory responses and expression of inflammatory cytokines in the tissue (25). Shin et al. have shown that exosomes-resembling sEVs derived from human adipose tissue-derived MSCs may significantly restore the epidermal barrier function in AD by inducing *de novo* synthesis of ceramides and modulating multiple gene expression programme, including the effects on differentiation of keratinocytes, lipid metabolism, cell cycle, and immune response (202). MSC-derived sEVs were shown to inhibit local inflammatory reaction and reduce tissue damage in atopic eczema (203). Hence, the evidence suggests that MSC-derived sEVs could potentially offer a promising cell-free therapeutic option for AD patients.

Contact allergy and contact sensitization is a common form of a delayed type hypersensitivity to small contact allergens. Contact allergy often develops after repeated or prolonged topical exposure to a particular sensitizing agent (204–206). Nazimek et al. have shown that intravenous administration of syngeneic mouse red blood cells leads to the EV generation that suppresses directed delayed type hypersensitivity in a miRNA-150-dependent manner; specifically, the syngeneic mouse red blood cell-derived EVs decreased T cell activation and enhanced their apoptosis (207). Similarly, human umbilical cord MSC-derived EVs were demonstrated to ameliorate and prevent the pathology of contact hypersensitivity in mice. Specifically, these EVs had a suppressive effect on both CD8^+^ cytotoxic cells and CD4^+^ Th1 cells, including the effect on TNF-α and IFN-γ production, induction of Tregs and the level of secreted IL-10 (208).

### Food Allergy and Allergic Inflammation in the Gastrointestinal Tract

Food allergy is a manifestation of an abnormal immune response to food or food additives (209) which is a complex process involving multiple cellular and molecular mechanisms. It has been shown that early exposure to allergens *via* the gastrointestinal route promotes tolerance (210, 211). It is not clear how much EVs are involved in this process, however, animal models suggest that there could be some contribution. Specifically, intestinal epithelial cells (IECs) subjected to OVA release sEVs that carry IL-10 and OVA/MHC class II complexes recognized by OVA-specific TCR-bearing CD4^+^ T cells. Here, OVA-specific CD4^+^ T cells represent type 1 Tregs, produce IL-10 and show immune suppressive effects on effector T cell proliferation. The proposed mechanism involved the role of vasoactive intestinal peptides, which seemed to be required for this effect (212, 213). Furthermore, Treg bias has been also observed following a sEV-mediated transfer of food allergens into the mesenteric lymph nodes (MLNs) of mice, in contrast to a direct transfer of those allergens, which promoted Th2 responses (214); the results also highlighted the role of exosomal integrin αvβ6 as a protective molecule. Finally, given that the diverse composition of the gut microbiome has been shown to be critical in food allergy prevention (215), antigen and mediator transfer *via* EVs secreted by IECs may be also involved in the elimination of pathogenic bacteria to prevent intestinal dysbiosis (146).

## Clinical Perspectives

Growing attention has been given to EVs as mediators in both physiological conditions and pathology, including the role in allergic diseases. Extensive research has been carried out showing the capacity of EVs to regulate homeostasis and immune functions in the allergic microenvironment. Alterations in exosomal content in allergic conditions have been shown to distinguish between physiological and diseased states suggesting the potential use of sEVs as biomarkers in the search of diagnostic tools for allergic diseases, for example in asthma phenotype subgrouping (216). Naturally-occurring sEVs can be also potentially used as drugs themselves, supporting healing process, e.g. MSC-derived sEVs participating in wound healing and regeneration of the lung tissue; this highlights the possible use of these sEVs in allergic airway remodeling (158, 202, 217). Several studies have proposed treatment strategies in animal models of allergic disease as summarized in **Table 2**. There are also examples of the use of sEVs as compound carriers are now being investigated as a naturally derived drug delivery systems (DDSs) with a favorable biocompatibility profile, but sEVs can be also potentially used to deliver non-drug anti-inflammatory agents including miRNAs (e.g. let-7-miRNAs). Indeed, there have been already several clinical trials in the past and more are now ongoing which investigate a potential of using EVs for the benefit of allergic patients (**Table 3**).

**Table 2:** Preclinical models using EVs for allergy treatment in animals.

Study Title	Conditions	Outcomes	Reference
Exosomes from Bronchoalveolar Fluid of Tolerized Mice Prevent Allergic Reaction	Allergy	BALF-derived exosomes induce tolerance and protection against allergic sensitization in mice.	Prado et al, 2008 (191)
Proinflammatory role of epithelial cell-derived exosomes in allergic airway inflammation	Asthmatic inflammation	IL-13 treated epithelial cell-derived exosomes induce enhanced proliferation and chemotaxis of undifferentiated macrophages in the lungs during asthmatic inflammatory conditions.	Kulshreshtha et al, 2013 (20)
Selective release of miRNAs via extracellular vesicles is associated with house dust mite allergen-induced airway inflammation	Allergic airway inflammation	Selective sorting of Th2 inhibitory miRNAs into airway secreted EVs and increase release to the airway is involved in the pathogenesis of allergic airway inflammation.	Gon et al, 2017 (218)
Exosomes derived from human adipose tissue-derived mesenchymal stem cells alleviate atopic dermatitis	Atopic dermatitis	Intravenously or subcutaneously injected human adipose tissue-derived MSC-derived exosomes ameliorate AD in an *in vivo* mouse model.	Cho et al, 2018 (25)
Extracellular vesicles from mesenchymal stem cells prevent contact hypersensitivity through the suppression of Tc1 and Th1 cells and expansion of regulatory T cells	Allergic contact dermatitis	Human umbilical cord-derived MSC-EVs prevent the pathology of contact hypersensitivity by inhibiting Tc1 and Th1 immune responses and inducing the Tregs phenotype *in vivo* and *in vitro*.	Guo et al, 2019 (208)
Small extracellular vesicles derived from human mesenchymal stromal cells prevent group 2 innate lymphoid cell-dominant allergic airway inflammation through delivery of miR-146a-5p	Allergic rhinitis (patients)	MSC-sEVs prevent ILC2-dominant allergic airway inflammation through miR-146a-5p.	Fang et al, 2020 (193)
	ILC2-dominant asthma (mouse model)		
Exosomes from Human Adipose Tissue-Derived Mesenchymal Stem Cells Promote Epidermal Barrier Repair by Inducing de Novo Synthesis of Ceramides in Atopic Dermatitis	Atopic dermatitis	Human adipose tissue-derived MSC-exosomes effectively repair defective epidermal barrier functions in atopic dermatitis.	Shin et al, 2020 (202)
Syngeneic red blood cell-induced extracellular vesicles suppress delayed-type hypersensitivity to self-antigens in mice	Delayed-type hypersensitivity	Intravenous delivery of syngeneic mouse red blood cells that is mediated by EVs in a miRNA-150-dependent manner suppresses delayed-type hypersensitivity.	Nazimek et al, 2020 (207)
	Contact hypersensitivity		
Intranasal delivery of MSC-derived exosomes attenuates allergic asthma *via* expanding IL-10 producing lung interstitial macrophages in mice	Allergic asthma	Intranasally delivered MSC-derived exosomes inhibit allergic asthma in mice.	Ren et al, 2020 (219)
Epithelial exosomal contactin-1 promotes monocyte-derived dendritic cell–dominant T-cell responses in asthma	Airway allergic models	Epithelial contactin-1 in exosomes is a critical player in asthma pathology.	Zhang et al, 2021 (181)
	Asthma		

**Table 3:** Registered clinical trial investigating the feasibility of using EVs in allergic patients.

Study Title	Conditions	Interventions	Locations	Identifier
Non-coding RNAs Analysis of Eosinophil Subtypes in Asthma	Allergic Asthma, Severe Eosinophilic Asthma	Biological: Dermatophagoides pteronyssinus allergen	Lithuanian University of Health Sciences, Pulmonology Department Kaunas, Lithuania	NCT04542902
		Procedure: Blood sampling, Procedure: Bronchial challenge with allergen		
Effectiveness of Qufeng Shengshi Fang on Treatment of Allergic Rhinitis	Rhinitis, Allergic, Perennial	Drug: Qufeng Shengshi Fang and Loratadine, Drug: Loratadine	Peking Union Medical College Hospital traditional Chinese medicine department Beijing, Beijing, China	NCT02653339
Cohort Study of the Patterns of Microvesicles in the Serum of Participants With Atopic and Non-atopic Asthma	Asthma, Allergies	Biological: tumor derived microparticles, Drug: cisplatin	The Ohio State University Medical Center Columbus, Ohio, United States	NCT00700726
Influence on Human Bronchial Epithelial Cells Smoker Extracellular Vesicles Influence on Human Bronchial Epithelial Cells	Smokers Human Bronchial Epithelial Cells Lung Pathogenesis Biomarkers	Diagnostic Test: Broncho Alveolar Lavages	HôpitalSaint-Philibert, Lomme, France	NCT03608293
Phase I/IIa Study on Chitin Microparticles in Subjects Suffering From Allergic Rhinitis	Seasonal Allergic Rhinitis	Drug: Chitin microparticles by nasal route	Hammersmith Medicines Research, London, United Kingdom	NCT00443495
Exploratory Study of the Cutaneous Penetration of Biodegradable Polymeric Microparticles in Atopic Dermatitis (MicroIskin)	Atopic Dermatitis	Drug: Biodegradable and biocompatible polymeric microparticles containing a fluorochrome applied to the skin followed by a skin biopsy	Regional University Hospital Besançon, France	NCT02369432
Impact of Narrowband UVB Phototherapy on Systemic Inflammation in Patients With Atopic Dermatitis	Atopic Dermatitis	Other: Narrow band UVB treatment, (NB-UVB)	The Rockefeller University New York, New York, United States	NCT03083730
Trial on Vascular Inflammation in Atopic Dermatitis	Atopic Dermatitis Vascular Inflammation Coronary Atherosclerosis	Other: FDG-PET Scan Other: MDCT, Other: biopsy and blood collection	Innovaderm Research Inc Montreal, Quebec, Canada	NCT02926807
Role of Macrophage in immune-modulation by mesenchymal stem cell derived exosome in asthma	Respiratory diseases	Primary indicator: PD-L1, Immuno-suppression capacity of regulatory T cell	Sun Yat-Sen Memorial Hospital, Sun Yat-Sen University	ChiCTR2000031122

In summary, non-immune cell-derived EVs contribute to allergic inflammation in the tissue location and potentially systemically; they have a great potential to become a valuable diagnostic option as well as a novel target for allergy therapy. Such EVs are slowly introduced into the clinic within the setting of clinical trials which investigate the feasibility of such an approach.

## Author Contributions

LH and DG-O wrote the paper. EC, DG-O, and LH prepared the figures. All authors contributed to the article and approved the submitted version.

## Funding

This work was supported by POIR.04.04.00-00-21FA/16-00 project, carried out within the First TEAM programme of the Foundation for Polish Science co-financed by the European Union under the European Regional Development Fund and National Science Centre, Poland - UMO-2016/23/P/NZ6/04056, this project has received funding from the European Union’s Horizon 2020 research and innovation programme under the Marie Skłodowska-Curie grant agreement No. 665778, as a part of POLONEZ Fellowship.

**Figure:**
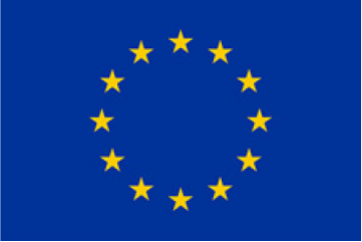


## Conflict of Interest

The authors declare that the research was conducted in the absence of any commercial or financial relationships that could be construed as a potential conflict of interest.

## Publisher’s Note

All claims expressed in this article are solely those of the authors and do not necessarily represent those of their affiliated organizations, or those of the publisher, the editors and the reviewers. Any product that may be evaluated in this article, or claim that may be made by its manufacturer, is not guaranteed or endorsed by the publisher.
